# Evaluative Observation in a Concealed Information Test

**DOI:** 10.3389/fpsyt.2019.00636

**Published:** 2019-09-11

**Authors:** Wolfgang Ambach, Birthe Assmann, Blanda Wielandt, Dieter Vaitl

**Affiliations:** ^1^Department Clinical and Physiological Psychology, Institute for Frontier Areas of Psychology and Mental Health, Freiburg, Germany; ^2^Lower Saxony Institute of Early Childhood Education and Development, Osnabrück, Germany; ^3^Institute for Frontier Areas of Psychology and Mental Health, Freiburg, Germany; ^4^Bender Institute of Neuroimaging, University of Giessen, Giessen, Germany

**Keywords:** Concealed Information Test, deception, mock crime, social stimuli, evaluative observation, orienting

## Abstract

The Concealed Information Test (CIT) is a valid method to detect hidden knowledge by means of psychophysiological measures. Concealing information is always a social behavior; yet, the role of social aspects has barely been investigated in recent CIT research favoring standardized, computer-based experiments. Evaluative observation is known to influence social behavior as well as physiological measures; examining the impact of evaluative observation on physiological responding in a CIT is the aim of this study. Sixty-three students completed a mock-crime and then underwent a CIT. In a between-subjects manipulation, half of the participants were observed through a camera and were faced with the real-time video of the experimenter watching them while completing the CIT. The other half completed the CIT without observation and video. Electrodermal activity, respiration line length, phasic heart rate, and finger pulse waveform length were registered. A specific questionnaire captured the individual fear of negative evaluation. Typical differential CIT responses occurred in both groups and with each measure. Contrary to expectations, differential CIT responses did not differ between groups. No modulatory influence of the fear questionnaire score on physiological responding was found. A ceiling effect, involving high attention and high motivation to avoid detection as well as high arousal in both groups due to the CIT procedure per se is discussed as explanation for these results, while the independence of the orienting reflex of social and motivational influence appears less likely in the light of previous literature.

## Introduction

### The Concealed Information Test

Among the manifold manifestations of deception, the concealment of information is a common type of deceptive behavior. For example, a culprit may exhibit this specific social behavior to appear uninvolved in a specific criminal act. The Concealed Information Test (CIT) is a scientific psychophysiological method to detect such intentionally hidden information. A systematic interrogation is hereby combined with a multi-channel physiological measurement. The CIT relies on the assumption that, if the examined subject is guilty, his or her physiological responses will differ between crime-related and crime-irrelevant information (1; for an overview, see 2), whereas an innocent examinee will not differentiate between crime-relevant and irrelevant details. The CIT consists of several multiple-choice questions each referring to another detail of the crime under investigation. Typically, there are four to five answer alternatives to each question; only one of these alternatives, the probe, refers to the critical detail. For example, if an envelope was stolen out of an office, a typical CIT question could be: “An office requisite has been stolen. Is this the stolen object?” This question is combined with a sequence of five pictures representing the respective answer alternatives, e.g., a picture of a) a pencil sharpener, b) an envelope, c) a highlighter, d) a stapler and e) a writing pad. In this example, the picture of the envelope (b) is the probe item; the other items are referred to as irrelevant. It is assumed that only guilty (knowledgeable) subjects will exhibit a different physiological response to the probe item. Unknowledgeable subjects, in contrast, will not exhibit different physiological responses to probe vs. irrelevant items; their response pattern will be unsystematic. The high validity of the CIT in the differentiation between guilty and innocent subjects was proven by a multitude of studies, as summarized by Ben-Shakhar and Elaad (3) or Meijer et al. (4).

The theory of the CIT strongly relies on cognitive aspects such as the orienting response (5, 6). Besides the orienting response, influences of motivation and emotion are discussed to play only a moderating role in the CIT when conducted in the laboratory. These influences might be greater when the CIT is applied in the field (2). Until now, only few studies focused on the influence of social factors, such as attention, intention, motivation, or emotion, on the CIT performed in the laboratory or in the field. Different authors have shown that motivation, intention, and emotion can affect response differences in the CIT (2). As an example, physiological response differences in the CIT have been shown to be enhanced by demanding a deceptive answer from the examinee, rather than demanding a truthful answer or no answer at all [e.g. Refs. (7, 8)], on the motivation to inhibit one’s own physiological arousal (9), and on the subject’s belief in the effectiveness of the physiological detection (10). Social aspects in turn are likely to have an impact on these factors. It seems worthwhile to study social and motivational influences on the CIT, as well as possible mediators of these influences, with more intensity and with a stronger focus on the effective mechanisms.

### Social Aspects and the CIT

Physiological functions and physiological responses are always influenced by the social context and by social stimuli. Zajonc (11) showed in his ‘social facilitation theory’, that the mere presence of another person enhances the physiological level of arousal. Perception of social gaze, which is evolutionary meaningful (12) is accompanied by specific subjective sensations and neurophysiological reactions (13). With respect to the CIT, which relies on physiological responding to specific stimuli, social influence has barely been investigated. Presumably, due to the desire to standardize CIT experiments as far as possible and also driven by the increasing use of computers in experiments, social aspects have played only a minor role in past CIT research. However, concealing knowledge from an interrogator is always a social act. Some decades ago, the social influence on the CIT was investigated in a small number of studies (14, 15). For instance, ethnic differences between subject and investigator, which were known to influence physiological parameters in general (16), enhanced physiological response differences in the CIT (15). Orne (14) broached the issue of possible differences between a friendly and an antagonistic investigator. However, these studies did not report on systematic experimental manipulations of social stimuli, social interaction, or social roles (17).

Particularly in real-life CIT examinations, as applied at a large scale and on a daily basis in Japan, social influences are inevitable and extensive (18). Emotion and motivation are supposed to be intense in an interrogation referring to a real crime. Elements of social interaction between examiner and examinee preceding and during the CIT could have an additive impact on a suspect’s motivation to remain undetected, on the intention to conceal, and on emotions like fear during the CIT. Notably, the contact between suspect and examiner in the CIT includes a wide spread of social elements: Social presence, eye contact, speech, sight, gestures, verbal interaction, and observation are just some examples. In the real-life CIT, these elements always occur and co-act in varying and hard to specify combinations. This makes it difficult to investigate them element by element. Experimentally varying single components of social influence in the laboratory is the best way to identify the components actually effective. Interestingly, a real-life interaction between examiner and examinee may vary in its positive vs. negative emotional impact. The same may hold for the positive vs. negative aspects of being observed and evaluated in real-life CIT examinations.

The influence of a first, specific set of social stimuli on physiological responding in the CIT was investigated in an earlier study (19). Employing the “voice of an interrogating person” asking the CIT questions, combined with presenting the image of the “face of an interrogating person” during the questions, lead to increased response differences. It remained open whether it was the acoustic questioning or the presented face, or their combination, that impacted the examinee’s physiological responses. Further, if a presented face in fact co-determines physiological responding in the CIT, then the specific connotation of that face for the subject becomes a central question. The impact of facial emotional expressions representing a virtual investigator on reaction times in a CIT was examined by Varga et al. (20). Interestingly, the mere presence of a virtual investigator’s neutral face led to an increase in overall but not differential reaction times, whereas emotional expression in this face was found to differentially increase reaction times to probe items. Most likely, a presented face or even a presented pair of eyes (21, 22) induces a feeling of being watched, controlled, or judged by another person (23), which should facilitate socially approved behavior while disapproved behavior, like deception, should become more difficult. For the present study, we focused on varying the “watching,” more precisely the “evaluative observation” component of social interaction.

### Evaluative Observation

Evaluative observation in a social context denotes a situation in which one person, while watching another, evaluates the behavior and performance of the other. Chapman (24) showed that the awareness of being observed evaluatively enhances arousal and raises the muscular tone. Cottrell et al. (25) showed that performing a task in front of an audience increases a person’s physiological arousal. Additionally, the presence of an audience enhanced dominant responses but the mere presence of others did not, which is contrary to Zajonc’s social facilitation theory (11). Other studies (26, 27) showed that anticipated evaluation of performance facilitated dominant responses but evaluation without awareness of being observed evaluatively did not. The finding that evaluative observation (with awareness) exerted social impact independently from social presence illustrates the importance and the possibility of experimentally separating individual components of social influence.

The same was hypothesized with respect to the CIT: We aimed to investigate the impact of evaluative observation on physiological responding in a CIT independently of the presence of another person. To create an environment, in which the subjects were observed evaluatively without the presence of another person, we decided to use a video camera. In this way, the experimenter could observe the subjects without being present in the experimental cabin. To ensure that the subjects were fully aware of being watched and critically evaluated, we installed, in addition to the according instructions, a second camera and presented a live video of the observing experimenter situated outside, on the subject’s screen.

Building upon the motivational impairment hypothesis (see 3, 28), we supposed that an examinee would show greater physiological response differences in the CIT when observed evaluatively. If this holds true, it might, on the one hand, help to enhance detection accuracy, and on the other hand, it might contribute to CIT theory by shedding light on the interplay of social influences, emotional-motivational factors, and physiological responding in the CIT.

To explore whether the impact of evaluative observation varies between subjects according to specific traits, we included the German version of the FNE (Fear of Negative Evaluation Scale) (29), called SANB (Skala Angst vor Negativer Bewertung; 30), which captures an individual’s fear of negative evaluation that is commonly understood as the cognitive component of social phobia. Following the finding of increased heart rate (HR) and palmar sweating in socially relevant situations (31), and also in view of earlier studies on trait influences on skin conductance (32) and reaction times (33) in the CIT, we supposed that people exhibiting a greater fear of negative evaluation would show not only increased overall responses but, due to a motivational impairment effect, also greater response differences between probe and irrelevant item types in the CIT. Additionally, we expected that people with a high fear of negative evaluation would also worry more about being detected, which in turn would facilitate detectability (34).

### Aim of the Present Study

1. This study focused on the influence of evaluative observation on physiological responding in a CIT. Two variants of the CIT, one condition, “with observation” and a second condition “without observation,” were manipulated between-subjects. Differential physiological responses were compared between groups. Greater differential responding was expected in the condition with evaluative observation for all physiological measures.
2. The study further investigated whether the differential physiological responses in the CIT are moderated by the individual fear of negative evaluation; therefore, the SANB questionnaire was included. With higher SANB scores, an enhancing influence of evaluative observation on physiological response differences was expected.

## Materials and Methods

### Subjects

Sixty-three healthy students (31 males, 32 females; mean age, 22.8 ± 2.4 years) voluntarily participated in the study. They were paid 12 Euros, with an additional incentive of 3 Euros. Data from one subject were discarded from evaluation because of a technical failure. The ethics committee of the German Psychological Society (DGPs) confirmed that the study met all ethical requirements (ID: WA122013).

### Procedure and Design

The experiment consisted of two parts, a mock-crime in an “office room” and a detection procedure in the “laboratory,” each guided by a different experimenter. The first experimenter welcomed the participant, explained the procedure, and accepted written informed consent. First, participants were told they had to perform a “special task” in an “office room,” for which they were asked to choose one out of five rolled-up instruction documents with different instructions. In fact, all documents contained the same mock-crime instruction. After the mock crime, participants walked over to the “laboratory” where the second experimenter expected them. This experimenter, in fact blind with respect to the mock-crime objects a particular participant had handled in the first part, was introduced as the person responsible for “detecting whether the subjects had stolen something in the office room or not.” After completing the CIT and a subsequent memory test, subjects filled in the SANB questionnaire (referring to trait anxiety of negative evaluation) before they were debriefed and released. Payment included the incentive of 3 Euros, regardless of a participant’s responding in the CIT.

Subjects were randomly assigned to either of two groups: Half of the subjects (i.e., the observation group; 31 valid data sets) underwent a CIT with particular emphasis on the fact that the experimenter was evaluatively observing them throughout the CIT; the other half (i.e., the no observation group; 31 valid data sets) underwent a CIT without evaluative observation.

For the observation group, a conspicuous camera was placed on top of the participant’s monitor, in addition to the inconspicuous camera generally surveying the experimental room from a corner. Written instructions stated that the experimenter’s aim was to find out “by means of precise observation *via* cameras, and by physiological measurement” whether the participant had stolen items from the office room or not. In three instances in advance of running the experiment, the experimenter himself explicitly mentioned these cameras and the importance of observation. To further direct attention to evaluative observation, the monitor-placed camera was adjusted again immediately before starting the CIT. Moreover, participants in the observation group viewed—between two subsequent item presentations—a full-screen live video of the experimenter critically watching them from outside and making written notices. The experimenter, while being watched *via* camera by the subjects, behaved in a pre-defined manner which emphasized attentive observation while excluding talking, laughing, gazing straight into the camera lens, as well as direct responding to the subject’s behavior. In fact, the experimenter filled in a score sheet continuously during the CIT, according to his or her conjectures about the items stolen by the individual participant.

In the no observation group, only the inconspicuous camera in the corner was installed, which was indispensable for conducting the experiment according to ethical standards; this camera was only briefly mentioned to the subjects as warranting they were in good hands. The experimenter’s aim was explained as “finding out by means of physiological measurement” whether the participant had stolen items from the office room or not.

### Mock-Crime Scenario

Alone and unwatched in an office room of the institute, subjects unrolled the document they had obtained from the first experimenter. By instruction, they had to remove (“steal”) nine objects from this room after having extensively viewed each of them. The choice of the nine objects, one from each category, was randomized and balanced across subjects. The nine object categories, each comprising five objects, were: key pendants, kitchen objects, boxes, office materials, cosmetics, wooden toy fruits, drink packages, playing cards, and plastic flowers.

Subjects were advised to collect all nine items in a suitcase, which they should keep closely to themselves throughout the remaining experiment. An amount of 3 Euros was hidden in one of the stolen objects (a box); later, this served as an incentive to “remain undetected” in the subsequent CIT.

### Concealed Information Test

The second experimenter conducted the CIT in the laboratory. For the so-called “physiological investigation,” recording devices were attached first. The CIT consisted of nine blocks referring to the nine item categories (e.g., key pendants, cosmetics). Each block comprised one question with five answer alternatives: the probe (“stolen”) item of each category and four corresponding irrelevant items, which were all unknown to the subjects.

CIT questions were presented acoustically with a pre-recorded male voice *via* speakers; “voice” questioning had turned out earlier as the more efficient variant (19). Different from the typical CIT wording, an active questioning format was chosen, which had also shown itself more efficient (35): Questions were, e.g., “Did you steal this cosmetic product from the administration room?” Each question was presented five times in sequence, each time directly followed by a different picture of one of the five answer alternatives.

The first item presented for each question served as buffer item; the according trials were discarded from analysis. Preceding each block, two neutral items were presented as distractors. The according questions referred to everyday objects that had to be identified (e.g., “Is this a slide projector?”). The two questions had to be answered correctly, one with “yes” and the other with “no” (in a pseudorandomized sequence) to keep participants alert and prevent them from answering automatically with “no” throughout the CIT. Responses to these neutral questions were not evaluated. Together with the two neutral questions preceding each category, the entire procedure resulted in a total of 63 item presentations. The main run was preceded by a training run consisting of two blocks, each with five neutral items.

**Figure 1:**
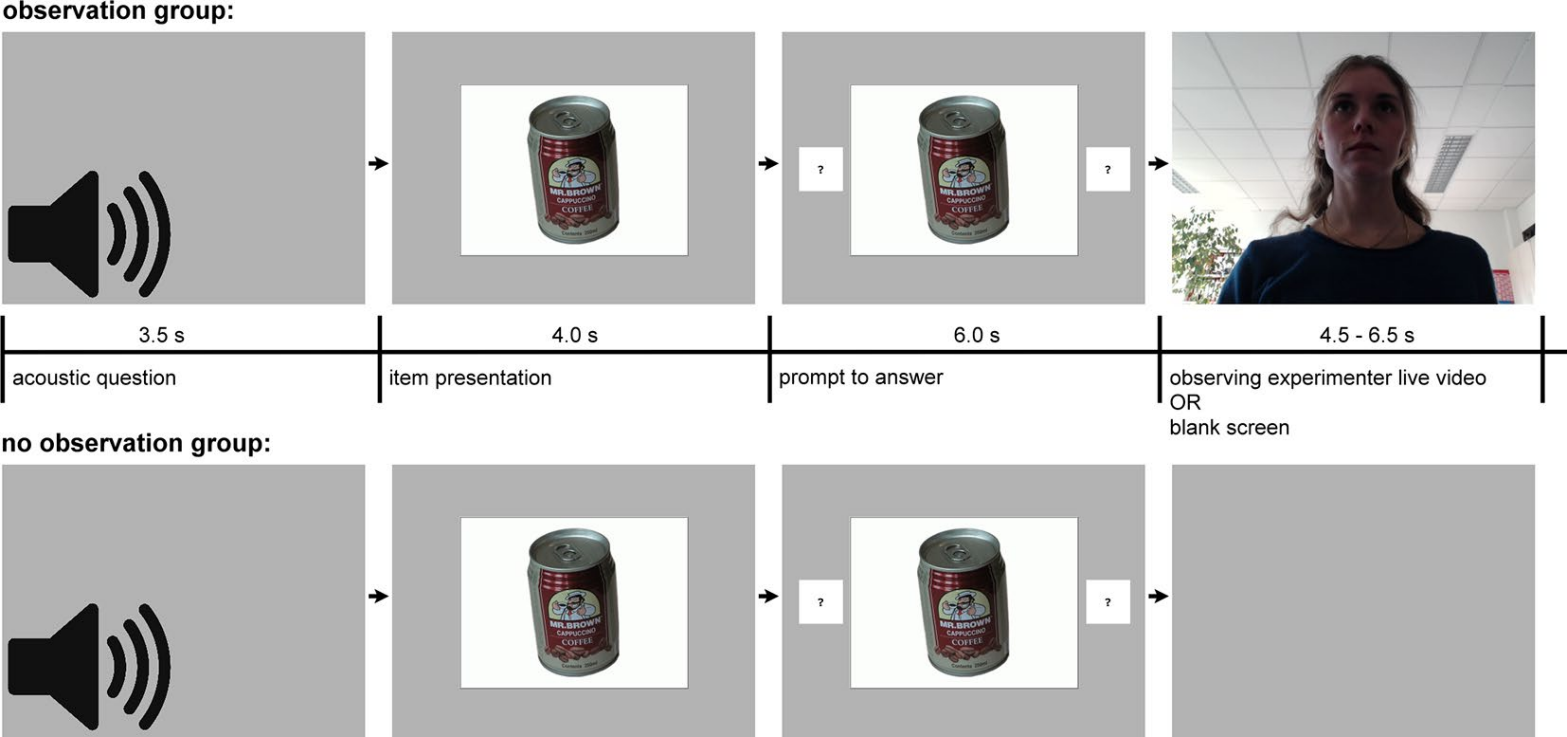
Temporal course of question and item presentation in the Concealed Information Test in the observation group and the no observation group. The acoustic question was presented first, CIT items appeared 3.5 s later, and fields with question marks succeeded 4.0 s thereafter. After the key press, a “yes” or “no” text (reflecting the subject’s answer) replaced the question marks.

**Figure 1** shows the intra-trial development of the screen for both groups. Acoustic question presentations were accompanied by 3.5 s of blank screen in both groups. Item pictures were presented for 10 s foveally on a 19-inch monitor at a distance of 90 cm, followed by the equally distributed 4.5 to 6.5 s display of either a live video showing the observing experimenter (in the observation group), or a blank screen (no observation group). Picture size was 14.3° by 10.7° of visual angle for the CIT items. Four seconds after an item was presented, two indication fields containing question marks appeared on either side of the item picture, this prompted the subjects to answer. Answers had to be given as quickly as possible by pressing one of the two response keys and by vocally responding with “yes” or “no.” Key assignment was balanced across subjects. Following the answer, the given “yes” or “no” replaced the question marks and remained visible on the screen as long as the item was presented. Subjects were told to hide their knowledge about the objects that had been stolen from the administration room, i.e., to deny all knowledge about probe items.

After subjects were disconnected from the leads, they underwent a memory test: All five pictures of each category were presented on the screen simultaneously, one item category after the other; subjects were asked to identify the item they had stolen within each category.

### Physiological Measures

The physiological recordings took place in a dimly lit and electrically and acoustically shielded experimental chamber (Industrial Acoustics GmbH, Niederkrüchten, Germany). Subjects sat in an upright position so that they could comfortably see the monitor and reach the keyboard. Temperature in the cabin was set to 21°C at the beginning of the first run, with an increase of maximum 2°C throughout the course of the experiment.

Skin conductance, respiratory activity, electrocardiogram (ECG), and finger plethysmogram were registered. Physiological measures were A/D-converted and logged by the Physiological Data System I 410-BCS manufactured by J&J engineering (Poulsbo, Washington). The A/D-converting resolution was 14 bit, allowing skin conductance to be measured with a resolution of 0.01 μS. All data were sampled with 510 Hz. Triggers indicating question onsets were registered with the same sampling frequency.

For skin-conductance recordings, standard Ag/AgCl electrodes (Hellige; diameter 0.8 cm), electrode paste of 0.5% saline in a neutral base (TD 246 Skin Resistance, Mansfield R&D, St. Albans, Vermont), and a constant voltage of 0.5 V were used. The electrodes were fixed at thenar and hypothenar sites of the nondominant hand. For registration of respiratory activity, two PS-2 biofeedback respiration sensor belts (KarmaMatters, Berkeley, California) with a built-in length-dependent electrical resistance were used. They were fixed at the upper thorax and the abdomen. ECG was measured with Hellige electrodes (diameter, 1.3 cm) according to Einthoven II. Finger pulse signal was transmitted by an infrared system in a cuff around the middle finger of the nondominant hand.

### Behavioral Measures

Subjects responded verbally as well as by pressing a key. Key presses indicating “yes” or “no” answers were time-logged, synchronized with the physiological measures and stored on the stimulus-presenting computer. Importantly, answers were delayed by 4 s in this study. After this delay, most stimulus processing and answer preparation can be assumed to be completed; in addition, it is rather easy to perform strategic manipulations by voluntarily controlling reaction speed after the delay. Therefore, behavioral data were not analyzed. CIT questions with at least one item answered incorrectly were discarded from the analysis, which resulted in a loss of 1.4% of the data.

### Questionnaire

As the last part of the experiment, participants filled in the SANB questionnaire. It comprises 20 items to assess the individual fear of negative evaluation as a trait variable. The sum scale was calculated from the raw data according to Vormbrock and Neuser (30).

### Data Processing

Skin conductance data from four subjects (two from the observation group, two from the no observation group) had to be discarded from the analysis because of electrodermal non-responding. Skin conductance reactions were assessed by a computerized method [see Refs. (7, 19, 36)] based on the decomposition of overlapping reactions as proposed by Lim et al. (37). This method was chosen, because two subsequent physiological reactions occurred with a short delay, due to the delay of 4 s between a question and the prompt to answer. With short interstimulus intervals, conventional trough-to-peak evaluation is inadequate (38) because the first of two reactions causes a diminishing bias in the estimation of the second one. The size of this bias is determined by the size of the first reaction and by the time interval between both reactions. Decomposition aims at overcoming this problem of overlapping electrodermal responses.

After optimizing model coefficients for each subject, all trials were evaluated by decomposing electrodermal activity (EDA) by use of each subject’s individual model coefficients. Then, magnitudes of all EDA responses that were elicited within a time window of 0.5 to 4.5 s after item presentation were additively combined to a first response (EDA_1). Magnitudes of EDA responses, which began between 4.5 and 8.5 s after item presentation, i.e., between 0.5 and 4.5 s after the subjects were prompted to answer, were additively combined to a second response (EDA_2). In addition, a combined response measure (EDA_sum) was calculated by adding both components per trial. For each time window, the decomposed responses were transformed into their equivalent amplitudes in µS according to each subject’s individual electrodermal response template.

Respiratory data were low-pass filtered (10 dB at 2.8 Hz); respiration line length (RLL) was automatically computed over a time interval of 15 s after trial onset. The RLL measure integrates information about frequency and depth of respiration. The method was derived from Timm (39) and modified by Kircher and Raskin (40). Respiratory data from nine subjects (four from the observation group, five from the no observation group) were discarded due to sensor problems. For analysis, raw scores from both respiratory channels were averaged.

ECG data obtained from one subject (from the observation group) had to be excluded from analysis because of technical failure. After notch filtering at 50 Hz, R-wave peaks were automatically detected and visually controlled. The R-R intervals were transformed into HR and real-time scaled (41). The HR during the last second before trial onset served as pre-stimulus baseline. The phasic HR (pHR) was calculated by subtracting this baseline value from each second-per-second poststimulus value. To extract the trial-wise information of the phasic HR, the mean change in HR within 15 s after trial onset, compared to the prestimulus baseline, was calculated [see Refs. (42, 43)].

Finger pulse waveform length (FPWL) data from four subjects (three from the observation group, one from the no observation group) had to be discarded from analysis because of insufficient signal quality. The FPWL within the first 15 s after trial onset was calculated from the finger pulse waveform and then subjected to further analyses (44). It comprises information about both HR and pulse amplitude.

To compare indicators of arousal between groups, we additionally computed the individual averages of non-standardized skin conductance level (SCL) and HR at trial onsets. The SCL and HR data were averaged over the last second before the onset of a CIT question, i.e., 3.5 to 4.5 s before item onset.

A within-subject standardization of measured values has been proposed by Lykken and Venables (45). Here, according to Ben-Shakhar (46), Gamer et al. (47), and Gronau et al. (48), the physiological measures are z-transformed for each subject and for each data channel. All probe and irrelevant trials (but neither neutral trials nor the first trials of each stimulus category) were used to calculate individual means and standard deviations. The z-transformed values were used in subsequent statistical analyses.

### Statistical Analysis

Statistical analyses were performed with SYSTAT, Version 13 (SYSTAT Software, Inc., Monte Carlo).

For each physiological measure, mean responses to probe vs. irrelevant items were compared using one-tailed t-tests (matched samples) separately for observation and no observation group. An additional t-test (two-tailed, independent samples) was performed to test whether the probe-minus-irrelevant response differences differed between groups. Cohen’s d was calculated as estimate of effect size (49, 50). To test for group effects on tonic physiological measures of arousal, means of SCL and HR were determined in the second preceding the acoustic question presentations, i.e., from 4.5 to 3.5 s before item onsets. To test between groups, two-tailed independent-samples t-tests were conducted on the basis of raw values. Significance level of all analyses was set to 0.05.

For identifying the fear of negative evaluation as a moderator of differential physiological responding in the CIT, correlation coefficients were calculated for the individual SANB sum scores and the individual standardized probe-minus-irrelevant response differences for each physiological data channel. Testing whether the individual SANB score is moderating the influence of evaluative observation on differential responding in the CIT was later dropped from analysis, after the influence of evaluative observation, *per se*, turned out insignificant.

## Results

### Memory Test

In the memory test, 98.6% of the probe items were identified correctly (97.8% in the observation and 99.3% in the no observation group). Categories with false identification of the probe item were entirely discarded from evaluation.

### Overview of Psychophysiological Measures

Preceding data standardization and test statistics, descriptive statistics based on raw scores are presented. **Table 1** summarizes means and standard errors of means of raw scores for each data channel separately for both groups.

**Table 1:** Means and standard errors of means (SEM) of raw scores for each data channel. Responses to *probe* and *irrelevant* items are listed separately for *observation* and *no observation* group.

	*Observation* group				*No observation* group			
	*Probe* items		*Irrelevant items*		*Probe* items		*Irrelevant items*	
	Mean	SEM	Mean	SEM	Mean	SEM	Mean	SEM
EDA_1 [nS]	262	47	139	24	298	60	132	23
EDA_2 (nS]	261	52	178	35	295	54	209	31
pHR [1/min]	−4.13	0.59	−1.58	0.36	−3.38	0.57	−1.59	0.29
RLL [arb. units]	2218	212	2517	229	2278	199	2645	204
FPWL [arb. units]	1416	149	1603	158	1702	190	1947	210

The physiological measures were first electrodermal response component (EDA_1), second electrodermal response component (EDA_2), phasic heart rate (pHR), respiration line length (RLL), and finger pulse waveform length (FPWL).

**Figure 2:**
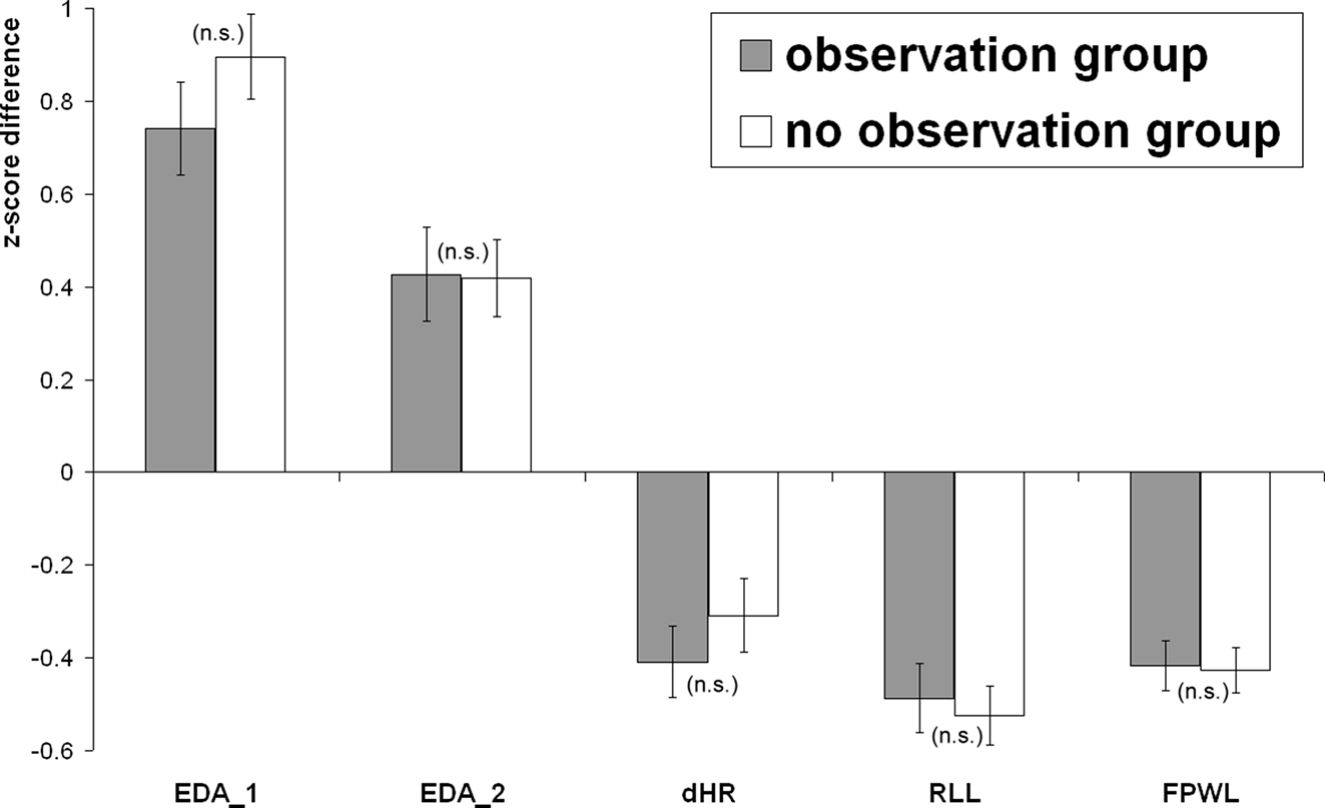
Differential responses (z-scores) to probe vs. irrelevant items: For the observation and the no observation group, standardized response differences are depicted for first electrodermal reaction (EDA_1), second electrodermal reaction (EDA_2), phasic heart rate (pHR), respiration line length (RLL), and finger pulse waveform length (FPWL). Error bars represent the standard error of the mean; the level of significance of the group difference is indicated by “n.s.” (not significant; p > 0.05).

**Figure 2** illustrates the differential responses to probe vs. irrelevant items for both groups. Response differences (z-scores) between probe and irrelevant trials are depicted for each of the physiological measures.

### Skin Conductance

**Figure 3:**
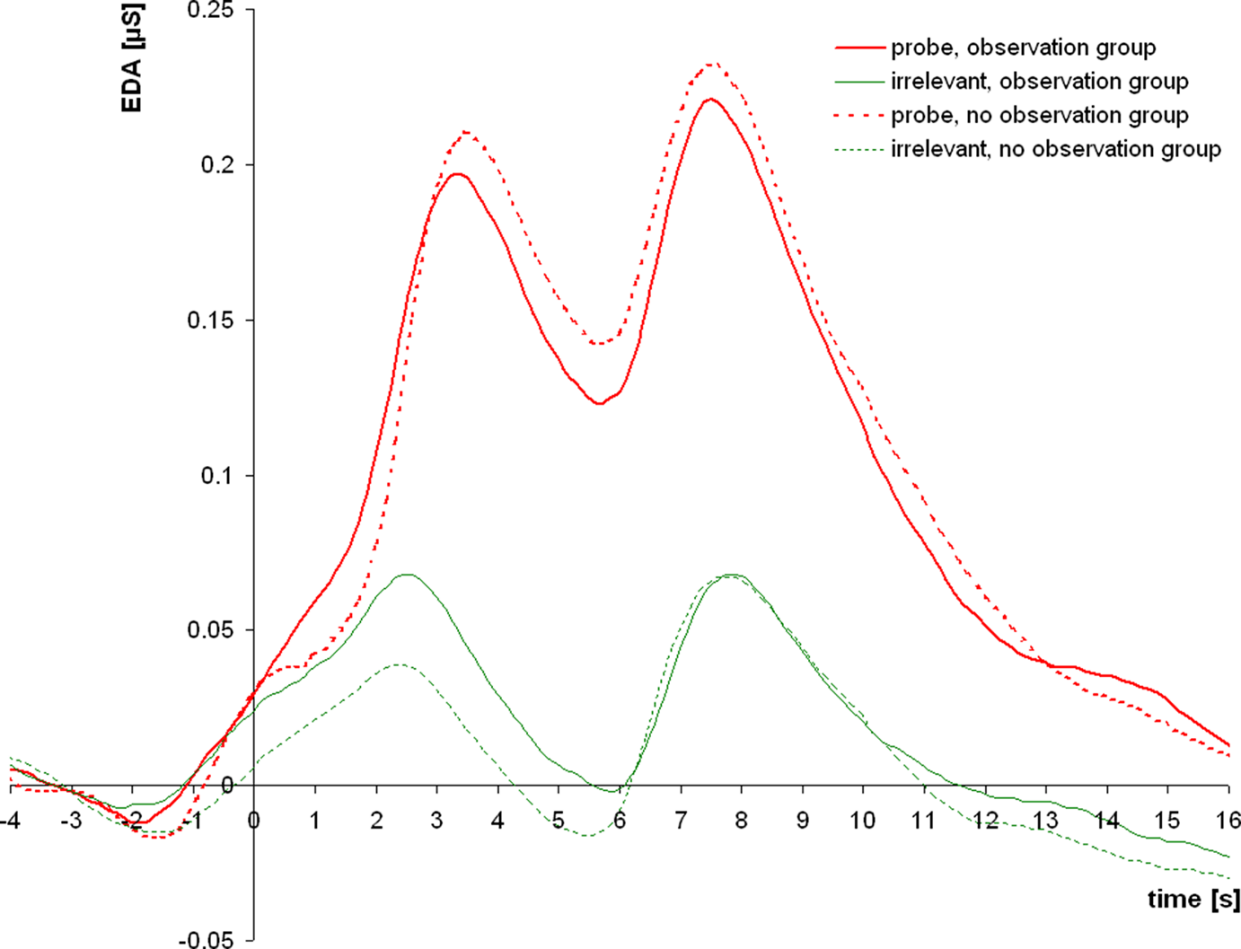
Grand means of skin conductance responses to probe and irrelevant items for the observation group and the no observation group. After a small initial response to the acoustic question presentation, two subsequent electrodermal responses of interest (EDA_1 and EDA_2) follow the item presentation and the prompt to answer, respectively.

**Figure 3** shows the averaged intra-trial course of skin conductance depicting grand means for trials with probe and irrelevant items separately for both groups. The grand means show two strong EDA response components with an onset and peak asynchrony of 4 s, which is in accordance with the 4-s delay between item onset and prompt to answer. Response amplitudes to probe items exceeded those to irrelevant items by far in both groups, with no apparent difference between groups. The additional EDA response, which was observed 3.5 s before the response to item onset, can be ascribed to the onset of the acoustic question presentation (which was the same for all items of a category).

EDA_1 responses were greater to probe than to irrelevant items in the observation group (t_26_ = 7.41; p < 0.001; d = 1.43) as well as in the no observation group (t_27_ = 9.85; p < 0.001; d = 1.86). The between-groups t-test for probe-minus-irrelevant EDA_1 response differences did not reveal a group difference (t_53_ = −1.41; p > 0.1).

EDA_2 responses were greater to probe than to irrelevant items in the observation group (t_26_ = 4.19; p < 0.001; d = 0.81) as well as in the no observation group (t_27_ = 5.07; p < 0.001; d = 0.96). The between-groups t-test for probe-minus-irrelevant EDA_2 response differences did not reveal a group difference (t_53_ = 0.07; p > 0.1).

Both EDA components were then additively combined in a single measure: EDA_sum. EDA_sum responses were also greater to probe than to irrelevant items in the observation group (t_26_ = 6.77; p < 0.001; d = 1.30) as well as in the no observation group (t_27_ = 8.38; p < 0.001; d = 1.58). Probe-minus-irrelevant response differences for EDA_sum did not differ between groups (t_53_ = −1.00; p > 0.1).

### Respiration

RLL values were smaller after probe than after irrelevant items in the observation group (t_26_ = −6.45; p < 0.001; d = −1.24) as well as in the no observation group (t_25_ = −8.12; p < 0.001; d = −1.59). Probe-minus-irrelevant response differences for RLL did not differ between observation and no observation group (t_51_ = 0.38; p > 0.1).

### HR

HR decelerations were more pronounced after probe than after irrelevant items in the observation group (t_29_ = −5.25; p < 0.001; d = −0.96) as well as in the no observation group (t_30_ = −3.84; p < 0.001; d = −0.69). Probe-minus-irrelevant response differences for pHR did not differ between observation and no observation group (t_59_ = −0.90; p > 0.1).

### Finger Pulse

FPWL values were smaller after probe than after irrelevant items in the observation group (t_27_ = −7.69; p < 0.001; d = −1.45) as well as in the no observation group (t_29_ = −8.68; p < 0.001; d = −1.58). The between-groups t-test for probe-minus-irrelevant FPWL differences did not reveal a difference between groups (t_56_ = 0.15; p > 0.1).

### Tonic Measures of Arousal

**Figure 4:**
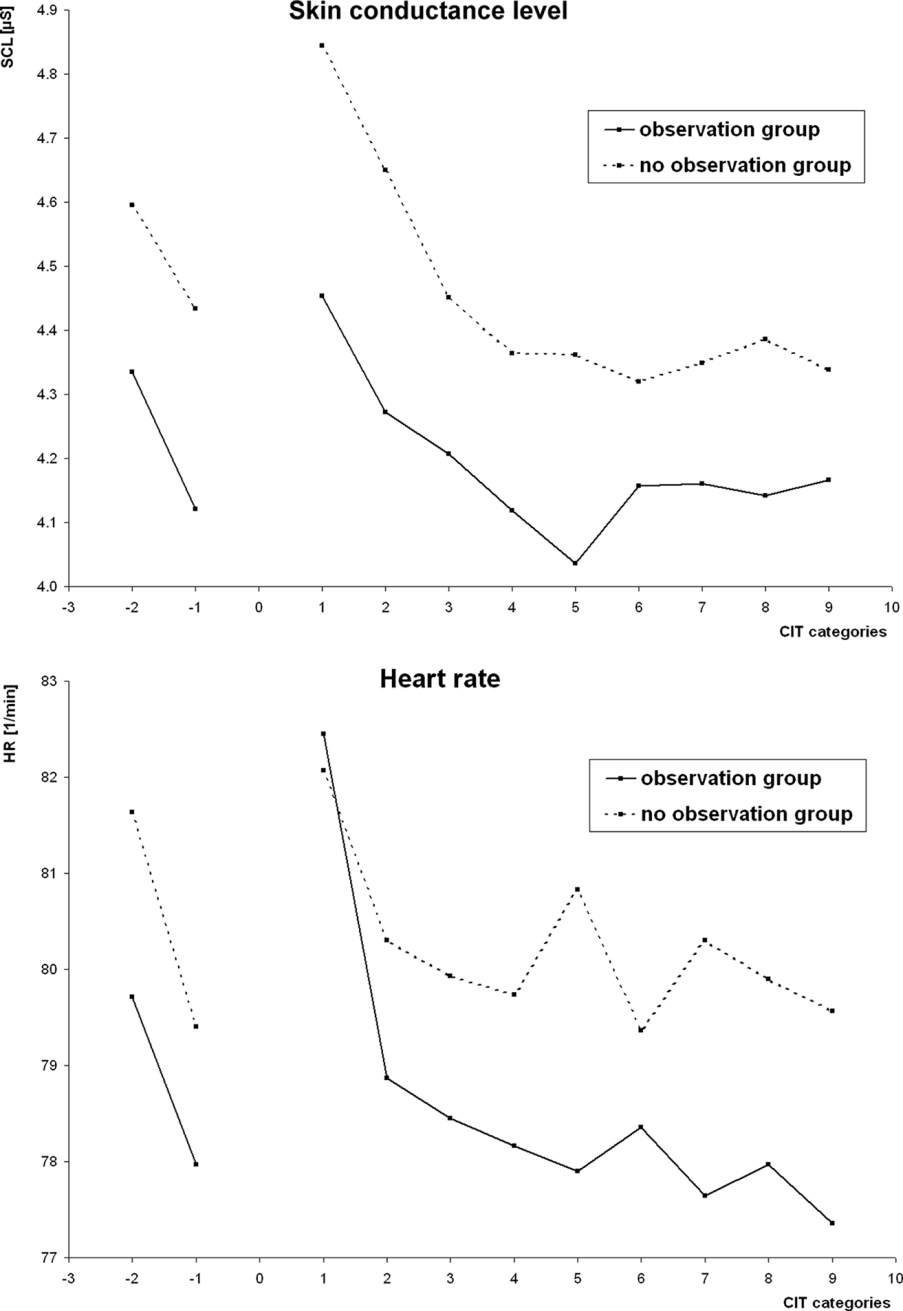
Skin conductance level (SCL) and heart rate (HR) as tonic measures of arousal. Grand means of raw values in the course of the experiment are depicted for the observation group and the no observation group. Data were collapsed within item categories; the first two data points of each plot reflect the training run.

As indicators of arousal, SCL and HR at trial onsets were analyzed. **Figure 4** depicts grand means of SCL (top) and HR (bottom) in the course of the experiment; data were collapsed over trials within categories; the first two data points of each subplot correspond to the two categories of the training run.

SCL appeared higher in the no observation group (4.64 ± 1.59 µS) than in the observation group (4.28 ± 1.66 µS). This was contrary to the expectation; yet, this difference was not statistically significant (t_53_ = 0.83, p > 0.1). Inspection of the raw data, see **Figure 4** (left), indicated that this result was due to an initially higher EDA level in the no observation group that was preserved throughout the entire examination. HR appeared higher in the no observation group (80.01 ± 9.14 bpm) than in the observation group (78.79 ± 11.24 bpm); yet, this difference was not statistically significant (t_59_ = 0.47, p > 0.1). The visual impression of a larger HR decrease over the experiment in the no observation group, see **Figure 4** (right), was not confirmed in a 2 (groups) × 9 (categories) ANOVA (F8 = 1.05; p > 0.1).

### SANB Questionnaire

The individual SANB sum scores were calculated. SANB data from the one participant precluded from physiological analysis were treated as missing data.

SANB sum scores were 45.68 ± 10.15 (median, 46) for female participants (n = 31) and 41.55 ± 9.58 (median: 41) for male participants (n = 31). This difference was not statistically significant (t_60_ = −1.65, p > 0.1). Scores for males as well as females markedly exceeded those reported originally for a student sample (median for females: 37, for males: 36; standard deviation unreported) (30). Data from males and females were then collapsed for further analyses.

SANB scores did not differ between observation and no observation group (43.84 ± 9.83 and 43.39 ± 10.34, respectively; t_60_ = −0.176, p > 0.1).

Correlation coefficients between individual SANB sum scores and probe-minus-irrelevant response differences for each physiological measure (EDA_sum, pHR, FPWL, and RLL) were calculated across groups as well as separately for the no observation and the observation group. Correlation coefficients for RLL were 0.31 across groups, 0.03 for the no observation, and 0.51 for the observation group. Here, the correlation across groups did significantly differ from zero before but not after Bonferroni correction for multiple testing (p = 0.024 and p > 0.1, respectively), whereas the correlation for the observation group did significantly differ from zero before as well as after Bonferroni correction (p = 0.003 and p = 0.036, respectively). Thus, differential respiratory responding in the CIT was found to be moderated by SANB sum scores in the observation group. For the other physiological measures, none of the corresponding correlations exceeded ±0.15; correspondingly, none of the correlations did significantly differ from zero (all p > 0.1, uncorrected).

## Discussion

The present study followed the idea that being observed evaluatively by an examiner during a CIT might enhance the examinee’s differential physiological responsiveness to probe vs. irrelevant items. A CIT condition “with observation,” in which subjects were observed *via* a conspicuous camera and presented with a live video of the experimenter watching them, was contrasted with a CIT “without observation.” A questionnaire on fear of negative evaluation was administered to explore a specific trait as possible moderator variable.

### Differential Responding in the CIT

Response differences between probe and irrelevant items were found for the electrodermal, the respiratory, and the cardiovascular measure. The observed effect sizes were in line with the large physiological response differences characteristic for the CIT (4). As an additional detail adopted from earlier studies, two components of electrodermal responding were separated, one after item presentation and one after the prompt to answer. Both components, with the first component exceeding the second with respect to effect size (cf. 7), mirrored the typical CIT response pattern. In line with a number of earlier studies (19, 44, 51), FPWL yielded larger effect sizes than pHR and RLL, but did not exceed those of EDA in this study. Yet, it has to be noted that effect sizes obtained after a restriction of recorded data sets to “valid” data sets, e.g., due to electrodermal non-responding, frequent extrasystoly, or insufficient pulse signal quality, should be interpreted with caution.

### The Impact of Evaluative Observation

The two CIT conditions with and without evaluative observation were administered in a between-subjects manipulation. Features differing critically between experimental conditions were the written CIT instructions, verbal instructions, and non-verbal behavior by the experimenter, presence of a conspicuous camera on top of the monitor, and presentation of a live videostream of the experimenter watching.

Contrary to the *a priori* expectation, none of the physiological measures exhibited a statistically significant enhancement of probe-vs.-irrelevant response differences in the observation group. The observed, insignificant group differences in the mean differential responses did, with the exception of pHR, not even meet the predicted direction. Hence, the main alternative hypothesis for this study, i.e., the assumption of an enhanced CIT effect under evaluative observation, was rejected.

### Explanations, Implications, and Limitations

At a first glance, one might suspect that the experimental manipulation was insufficient. The participants’ motivation to cope with the test and their prospect of success, known to affect differential responding in the CIT (52, 53), and also their attention during the CIT, might not have differed sufficiently between conditions. Tonic physiological measures at stimulus onsets, i.e., tonic HR and SCL, were analyzed to test for differences in arousal between conditions. The lack of a statistically significant difference in these measures between conditions principally supports the idea of an insufficient experimental manipulation. Also, the video of the experimenter was shown to participants only in between CIT questions, so that the time window of its impact may be discussed. On the other side, subjects’ verbal reports after the experiment (gathered unsystematically during debriefing) underlined that the experimental manipulation was visually impressive and psychologically effective. Particularly the real-time view of the experimenter watching was described by participants as challenging, reminding of the presence of an opponent, and thereby enhancing the motivation to hide the critical knowledge “without letting anything show.” The influence of arousal on the CIT effect, which gave rise to the analysis of the two tonic measures in this study, was recently summarized by Klein Selle et al. (9). Given that no arousal difference between groups was found in this study, also the interpretation of other group effects deserves caution.

The psychological difference between conditions might also have been diminished by the fact that there was always one camera present in the room, even in the condition without observation. This camera, which was indispensable for the ethical correctness of the experiment, was not conspicuous, not positioned centrally, and mentioned only briefly by the experimenter as “necessary to make sure you are alright.” Nevertheless, this camera might have been sufficient to make participants even in the no observation group feel that they were watched critically throughout the test. Although conceivable, this possible explanation did not find support in participants’ later verbal reports. In addition, the difference between groups in the written instructions might have led to instruction-induced effects differing between groups. Given that the interaction between participant and examiner is always complex, the different instructions might have confounded possible group effects.

Next, subjects in both experimental groups were motivated to pass the test without being detected. The incentive of three Euros probably strengthened this aim. Yet, experimental participants generally tend to exhibit such motivation, rather than absolving the CIT incuriously. Thus, participants of either group were highly motivated not to be detected in the CIT. It can then be questioned to what extent such high motivation can be even further enhanced by additional observation and evaluation. In case of a general high level of motivation, a ceiling effect is well conceivable, which might contribute to explaining the lack of a difference between conditions in differential physiological responding.

A similar kind of ceiling effect might be caused by the physiological recording. An examinee might fear that his feelings will be uncovered by this recording, independently of his control. The rather uncommon experience of being attached to a physiological recording device while being questioned might lead subjects to feel like “being watched” and “being evaluated” intensively, even without camera. If so, then it can be questioned to what extent the feeling of being observed evaluatively can still be enhanced by an additional, “visual” observation.

In addition, it has to be noted that CIT questions were presented acoustically in both conditions. In contrast to the text variant of question presentation, acoustic presentation was thought to entail greater physiological response differences (19), presumably by inducing a more “social” experience, perhaps emphasizing the need to actively conceal knowledge and increasing the difficulty of doing so without letting anything show. Thus, the acoustic variant of question presentation that was employed in both conditions used the more social and presumably more efficient stimuli. This might have contributed to a ceiling effect: Voice as a social stimulus might have augmented physiological responding even in the no observation (control) condition, so that further augmentation by additional social stimuli approached a limit.

The study did not include groups of innocent (unknowledgeable) participants. This design was chosen in favor of comparing differential physiological responding between the two experimental groups with a maximum test power in relation to the number of participants. As a consequence, classification statistics, which would have been based on simulated data only, were omitted in this study.

Summarizing, whatever mediator variables are joining social impact and physiological responding, these mediators might perhaps already be augmented to a near-maximum degree in the CIT variant that was used as control condition in this study. Then, additional social influence, which was thought to heighten these mediator variables, would hardly be able to induce further enhancement. It can be seen as one limitation of the present study that no “low arousal,” “low social impact,” or “low motivation” condition was included which might have left more space for enhancement of differential physiological responding between conditions.

A more theoretical attempt to explain the lacking influence of evaluative observation refers to the orienting reflex and its modulation. Bradley (54) suggested to regard the orienting response as embedded in motivational and attentional systems that are active and fluctuating within an individual. This view gave rise to the assumption that social stimuli and social interaction influence the orienting response to stimuli; it was hypothesized that also the differential response to stimuli of different subjective significance is sensitive to social influence. Perceiving orienting from a classical perspective [cf. Ref. (6)] and focusing on the correlation between features of the individual stimulus (novelty, intensity, and significance) and the corresponding physiological response, one might possibly start to doubt that psychophysiological responding in the CIT depends on social factors at all. The fact that results of this study did not just miss statistical significance but did not even exhibit a clear direction, might be interpreted as support for this viewpoint. However, given the above alternative explanations and given that this study was the first attempt to manipulate evaluative observation in a CIT, the primary implication of negative results cannot refer to theory before clearing out the conjectured limitations of this study.

A more conceptual limitation of this study refers to the process of evaluative observation, which according to Chapman (24) was initially regarded as one elementary component of social interaction to be distinguished, e.g., from mere presence (55). In the aftermath, observation, evaluation, and the way in which both were realized and combined in this study, are thought to have entailed a set of social components more complex than intended. Although experimenter instructions were meant to help standardize the interaction between subject and experimenter, this interaction presumably had remained complex and multi-faceted.

### Trait Aspects: The Fear of Negative Evaluation and the CIT

For male as well as for female participants, SANB average scores exceeded those of a student sample reported earlier (30). Due to the small number of samples reported in the literature, the source of this difference cannot be pinpointed. Temporal change is conceivable, but also a biased sample cannot be ruled out in this study. Higher scores, however, would be expected to lead to greater rather than lower sensitivity of participants to the manipulation of evaluative observation, so that a biased sample is unlikely responsible for the negative results.

Overall, differential physiological responding was not correlated with SANB scores and SANB scores did not interact with the experimental manipulation. Possibly, the trait fear of negative evaluation, which the SANB captures, is of limited relevance when information is concealed from a social counterpart. Being observed with the assumed aim to detect bodily and behavioral indicators of deception might be critically different from being evaluated by observation with respect to performance, correctness, adequateness, or other. The one specific exception to that, namely, the interactive influence of trait fear and evaluative observation on the respiration measure, tentatively points toward a specific sensitivity of fearful examinees to evaluative observation; yet, this finding deserves replication before conclusions can be drawn. Data collection in this study did not include a state measure of fear which might have been fruitfully related to the experimental manipulation and which might have helped to sharpen possible effects of trait fear of evaluative observation.

### Suggestions for Future Studies

Continuing the study of social influence on physiological responding in the CIT is necessary. Recent attempts to resume this earlier line of research [see, e.g., Ref. (15)] abandoned in favor of standardization of experiments revealed specific difficulties. Problems arise from the complexity of social interaction, per se, and the severity of disentangling and “isolating” the individual components of social stimuli and social action.

One line of future research might split the focus into two: Studies might investigate the direct influence of those mediator variables that are assumed to link social influence to CIT responding, while other studies might investigate how the manipulation of social stimuli and interaction affects these mediator variables.

From a CIT application perspective, however, research manipulating the social context, in which the CIT is performed, directly in CIT studies is presumably indispensable. The experimental manipulations of social conditions in these CIT studies should then become less complex. For example, evaluative observation might be decomposed into two components, observation and evaluation, which should then be manipulated independently. Taking our experimental setup as an example, the live video showing the experimenter that was employed to remind the participants of being watched might be replaced by a more uniform implementation of the manipulation pursuing the same objective, i.e., induce awareness of being observed during the CIT. Furthermore, replacing acoustic question presentation by a textual interrogation, but perhaps also replacing the active wording of CIT questions by a passive wording, might help to avoid a ceiling effect and thus allow for greater response differences between conditions. Some caution will be needed, however, to prevent such tailored experimental manipulations to become too artificial for an applied setting. With respect to the applied CIT, it has to be borne in mind that in present field applications of the CIT, e.g., in Japan, the examiner is usually present within the same room as the examinee. In this light, laboratory studies like the present one, in which details of the social context in the CIT are manipulated, might contribute first to our theoretical understanding of basic socio-psychophysiological correlations, and perhaps second to practical implications.

## Conclusions

Being observed and evaluated during the CIT with awareness but in the absence of a physical examinator did not enhance an examinee’s differential physiological responding in the test. Furthermore, the individual fear of negative evaluation by others did not moderate physiological CIT responses. Standardization of experiments and investigation of social action are essentially conflicting aims even today. To further examine influences of the social situation in which the CIT is performed on physiological responding, it is suggested to manipulate social stimuli and elements of social action at an even more elementary level in future studies.

## Ethics Statement

This study was carried out in accordance with the recommendations of “the German Science Foundation and the German Society for Psychology (DGPs),” with written informed consent from all subjects. All subjects gave written informed consent in accordance with the Declaration of Helsinki. The protocol was approved by the “ethics committee of the German Society for Psychology (DGPs).”

## Author Contributions

WA, BA, and DV were in charge of planning the study. BW was in charge of preparing stimulus material and recruiting participants. BW and WA were in charge of conducting the experiments. WA was in charge of data analysis. WA, BA, BW, and DV were in charge of writing the paper.

## Conflict of Interest Statement

The authors declare that the research was conducted in the absence of any commercial or financial relationships that could be construed as a potential conflict of interest.
